# Risk factors for recurrent variceal bleeding after TIPS in cirrhotic patients with esophagogastric variceal hemorrhage: a single-center retrospective study

**DOI:** 10.3389/fmed.2026.1951291

**Published:** 2026-09-30

**Authors:** Zhiliang Chen, Yi Gou, Wenke Li, Daowu Yu, Changjie Du, Jue Yuan

**Affiliations:** 1 Department of Hepatobiliary Surgery, The Affiliated Yongchuan Hospital of Chongqing Medical University, Chongqing, China; 2 General Practice School of Chongqing Medical University, Chongqing, China

**Keywords:** esophageal and gastric variceal bleeding, rebleeding, risk factors, shunt dysfunction, transjugular intrahepatic portosystemic shunt

## Abstract

**Background:**

Transjugular intrahepatic portosystemic shunt (TIPS) effectively reduces portal pressure but post-procedural rebleeding remains a major clinical challenge. Risk factors for rebleeding are not fully defined.

**Methods:**

This single-center retrospective cohort study included 114 cirrhotic patients who underwent TIPS for EGVB (2019–2024). Patients were categorized into rebleeding (*n* = 35) and non-rebleeding (*n* = 79) groups based on 1-year post-TIPS rebleeding. Clinical, laboratory, procedural, prior endoscopic treatment history and follow-up data were analyzed. Univariate and multivariate Cox proportional hazards models were used to identify risk factors. Cumulative rebleeding incidence was compared between serum sodium groups (<135 vs. ≥135 mmol/L) using Kaplan–Meier analysis with log-rank test.

**Results:**

The 1-year rebleeding rate was 30.7% (35/114). Multivariate Cox regression identified lower serum sodium (HR = 0.85; 95% CI: 0.77–0.93; *P* < 0.001) and shunt dysfunction (HR = 4.60; 95% CI: 2.21–9.58; *P* < 0.001) as independent risk factors for rebleeding. Stent type was not independently associated after adjustment. The low sodium group (<135 mmol/L) showed a significantly higher cumulative rebleeding incidence than the high sodium group (log-rank *P* = 0.041). Conclusion: Serum sodium preoperatively and post-TIPS shunt dysfunction are independent risk factors for 1-year rebleeding. Optimizing serum sodium preoperatively and implementing regular surveillance for shunt dysfunction may help reduce rebleeding risk in these patients.

## 1 Introduction

Transjugular intrahepatic portosystemic shunt (TIPS) effectively reduces portal pressure by creating a shunt between the portal and hepatic veins, and has become a standard treatment for esophageal and gastric variceal bleeding (1). It is primarily indicated for acute variceal bleeding refractory to endoscopic and/or pharmacological therapy, as well as for secondary prophylaxis of variceal bleeding (2, 3). TIPS is also effective for refractory ascites and hepatorenal syndrome (4–6). Since its first clinical application in 1969, TIPS technology has continuously evolved, and in the modern era, hemostasis is achieved in over 90% of cases (7, 8). Previous studies have demonstrated that TIPS significantly improves rebleeding-free survival compared with endoscopic therapy (9–12).

Despite these advances, portal hypertensive rebleeding after TIPS remains a major clinical challenge, with reported 1- to 2-year rebleeding rates ranging from 6% to 40% (13, 14). Procedural factors, such as stent type and adjunctive embolization, have been extensively investigated. Covered stents improve shunt patency and reduce rebleeding compared with bare-metal stents (15, 16). However, the benefit of concomitant variceal collateral embolization remains controversial, with some studies reporting reduced rebleeding rates and others showing no significant advantage (14, 17). Beyond procedural variables, patient-related factors are increasingly recognized as important predictors. The ALTA multicenter study identified higher MELD-Na score and the presence of portal hypertensive gastropathy on pre-TIPS endoscopy as independent risk factors for rebleeding within 1 year (18). Nevertheless, the predictive value of other clinical indicators, such as preoperative serum sodium and Child-Pugh grade, has not been fully validated.

Therefore, this study aimed to retrospectively analyze clinical data from patients with EGVB who underwent TIPS at a single center over a 5-year period, and to evaluate the impact of demographic characteristics, laboratory parameters, and procedural factors on postoperative rebleeding, in order to identify high-risk patients and inform individualized management strategies.

## 2 Materials and methods

### 2.1 Study population

This was a single-center retrospective cohort study. Patients with cirrhosis who underwent TIPS for esophageal and gastric variceal bleeding (EGVB), between January 1, 2019, and December 31, 2024, were retrospectively identified through the electronic medical record system (approval number: 2026EC0123) which waived the requirement for informed consent for study participation; all patients had provided written informed consent for the TIPS procedure as part of routine clinical care.

Inclusion criteria were as follows: (1) age 18–80 years; (2) confirmed diagnosis of cirrhosis with at least one documented episode of EGVB; (3) underwent TIPS for EGVB, with successful stent placement and immediate post-procedural angiography confirming stent patency; (4) complete clinical data, including preoperative baseline characteristics, intraoperative records, and postoperative follow-up data; and (5) signed informed consent for the TIPS procedure from the patient or their family members as part of routine clinical practice. Exclusion criteria were as follows: (1) non-cirrhotic portal hypertension (e.g., cavernous transformation of the portal vein, Budd-Chiari syndrome); (2) prior history of TIPS or other portosystemic shunt surgery; (3) severe dysfunction of vital organs, including the heart, lungs, or kidneys; (4) pregnancy; and (5) incomplete clinical data or loss to follow-up. A total of 114 patients were ultimately included in the analysis.

### 2.2 Data collection

Data were extracted through retrospective review of electronic medical records, including the following: Demographic and clinical characteristics: sex, age, etiology of liver cirrhosis (classified as viral hepatitis, alcohol-related, or other causes), Child-Pugh grade, and history of prior endoscopic treatment for esophagogastric varices (including endoscopic variceal ligation, sclerotherapy, or tissue glue injection). Laboratory parameters: serum sodium, albumin, total bilirubin (TBil), prothrombin time (PT), international normalized ratio (INR), creatinine, alanine aminotransferase (ALT), aspartate aminotransferase (AST), blood ammonia, hemoglobin (Hb), and platelet count (PLT). All laboratory values were obtained from the most recent preoperative assessment. The MELD score was calculated using the standard OPTN formula without an upper limit. Procedural data: TIPS indication, type of stent (covered vs. bare-metal), stent diameter, whether combined embolization was performed, and pre- and post-TIPS portosystemic pressure gradient (PPG). The covered stents used were primarily the Angiomed Covera™ Vascular Covered Stent system (models FVM07040 for 7 mm and FVM08040 for 8 mm; Angiomed GmbH & Co. Medizintechnik KG, Karlsruhe, Germany), and the bare stents were from the Cook Zilver® family (models ZIV6-125-7-8.0 for 7 mm and ZIV6-125-8-8.0 for 8 mm; Cook Ireland Ltd.). All procedural data were derived from TIPS operative reports. Given the retrospective nature of this study, decisions regarding stent type, target post-TIPS PPG, and the use of adjunctive embolization were made at the discretion of the operator based on clinical circumstances, with no standardized protocols applied. Follow-up data: occurrence and timing of rebleeding, shunt dysfunction, hepatic encephalopathy, and death, including the cause of death where available.

### 2.3 Grouping and study endpoints

Rebleeding after TIPS was defined as portal hypertensive bleeding occurring within 1 year postoperatively (18). According to the Baveno VII consensus (19), rebleeding was specifically defined as any of the following occurring more than 48 h after TIPS: (1) endoscopically confirmed rupture of esophageal and/or gastric varices, with active bleeding or adherent clot; (2) clinical presentation of hematemesis and/or melena, with endoscopic confirmation of variceal bleeding; or (3) upper gastrointestinal bleeding after excluding other causes such as peptic ulcer disease or portal hypertensive gastropathy. Based on this definition, patients were divided into a rebleeding group (*n* = 35) and a non-rebleeding group (*n* = 79). The primary endpoint was the occurrence of gastrointestinal rebleeding within 1 year after TIPS. Secondary endpoints included all-cause mortality, hepatic encephalopathy, and shunt dysfunction. Shunt dysfunction was defined as meeting any of the following criteria (20): (1) color Doppler ultrasound showing absence of blood flow or reversal of flow direction within the shunt; or (2) ultrasound findings of shunt flow velocity <90 cm/s or >250 cm/s, or a change of >50 cm/s compared with the previous examination, accompanied by portal venography confirming >50% stenosis of the shunt and a rise in portal pressure to or above the preoperative level.

### 2.4 Statistical analysis

All data processing and statistical analyses were performed using SPSS version 27.0 (IBM Corp., Armonk, NY, USA). Continuous variables with a normal distribution were expressed as mean ± standard deviation (SD), while those with a skewed distribution were expressed as median with interquartile range (IQR). Categorical variables were presented as frequencies and percentages. For intergroup comparisons, the independent samples t-test was used for normally distributed continuous variables, the Mann–Whitney U test for skewed continuous variables, and the chi-square test or Fisher's exact test for categorical variables or proportions. Due to the extreme imbalance in sample distribution (e.g., 94% of patients underwent combined embolization), variables with severely unbalanced group sizes were excluded from the regression analysis to avoid unreliable statistical inferences. Univariate analysis was performed using the Cox proportional hazards regression model to calculate the hazard ratio (HR) and 95% confidence interval (CI) for each variable. Shunt dysfunction was treated as a time-dependent covariate using the counting process approach, with patients split into multiple intervals according to the occurrence time of dysfunction, coded as 0 before and 1 after occurrence, to eliminate immortal time bias. Variables with a *P*-value < 0.05 in the univariate analysis were subsequently entered into a multivariate Cox regression model using stepwise selection to identify independent risk factors. All tests were two-sided, and a *P*-value < 0.05 was considered statistically significant. For sensitivity analysis, patients were stratified into a low sodium group (<135 mmol/L) and a high sodium group (≥135 mmol/L) using a cutoff value of 135 mmol/L. Kaplan–Meier survival curves were generated using R software (version 4.5.2, R Foundation for Statistical Computing, Vienna, Austria), and intergroup comparisons were performed using the log-rank test.

## 3 Results

### 3.1 Baseline characteristics

Between January 1, 2019, and December 31, 2024, a total of 139 patients with cirrhosis who underwent TIPS for esophageal and gastric variceal bleeding (EGVB) at the Department of Hepatobiliary Surgery, Yongchuan Hospital of Chongqing Medical University, were initially screened. After excluding 25 patients (2 deaths due to postoperative intra-abdominal hemorrhage, 1 death due to postoperative pulmonary infection, 4 unsuccessful procedures, 5 lost to follow-up, 11 with a history of prior TIPS, and 2 with incomplete clinical data), 114 patients were ultimately included in the analysis (Figure 1). Among the 114 patients, 80 (70.2%) were male and 34 (29.8%) were female, with a mean age of 55.8 ± 10.3 years. The most common etiology of liver cirrhosis was viral hepatitis (72 patients, 63.2%), followed by other causes (22 patients, 19.3%) and alcohol-related liver disease (20 patients, 17.5%). Regarding Child-Pugh grade, 24 patients (21.1%) were classified as grade A, 73 (64.0%) as grade B, and 17 (14.9%) as grade C. The median MELD score was 10 (IQR: 9–14). Covered stents were used in 81 patients (71.1%), and bare-metal stents in 33 patients (28.9%). Combined embolization was performed in 107 patients (93.9%). The mean preoperative PPG was 30.1 ± 6.0 mmHg, and the mean absolute reduction in PPG (*Δ*PPG) was 15.0 ± 4.6 mmHg. Compared with the non-rebleeding group, the rebleeding group had significantly lower serum sodium levels (136.0 ± 3.0 mmol/L vs. 138.5 ± 4.1 mmol/L, *P* = 0.001), a significantly higher proportion of bare-metal stent use (42.9% vs. 22.8%, *P* = 0.029), and a significantly higher incidence of shunt dysfunction (37.1% vs. 7.6%, *P* < 0.001). No statistically significant differences were observed between the two groups in terms of age, sex, prior endoscopic treatment history, etiology of liver cirrhosis, Child-Pugh grade, preoperative PPG, absolute reduction in PPG, or MELD score (all *P* > 0.05). Detailed baseline characteristics are presented in Table 1.

**Figure 1:**
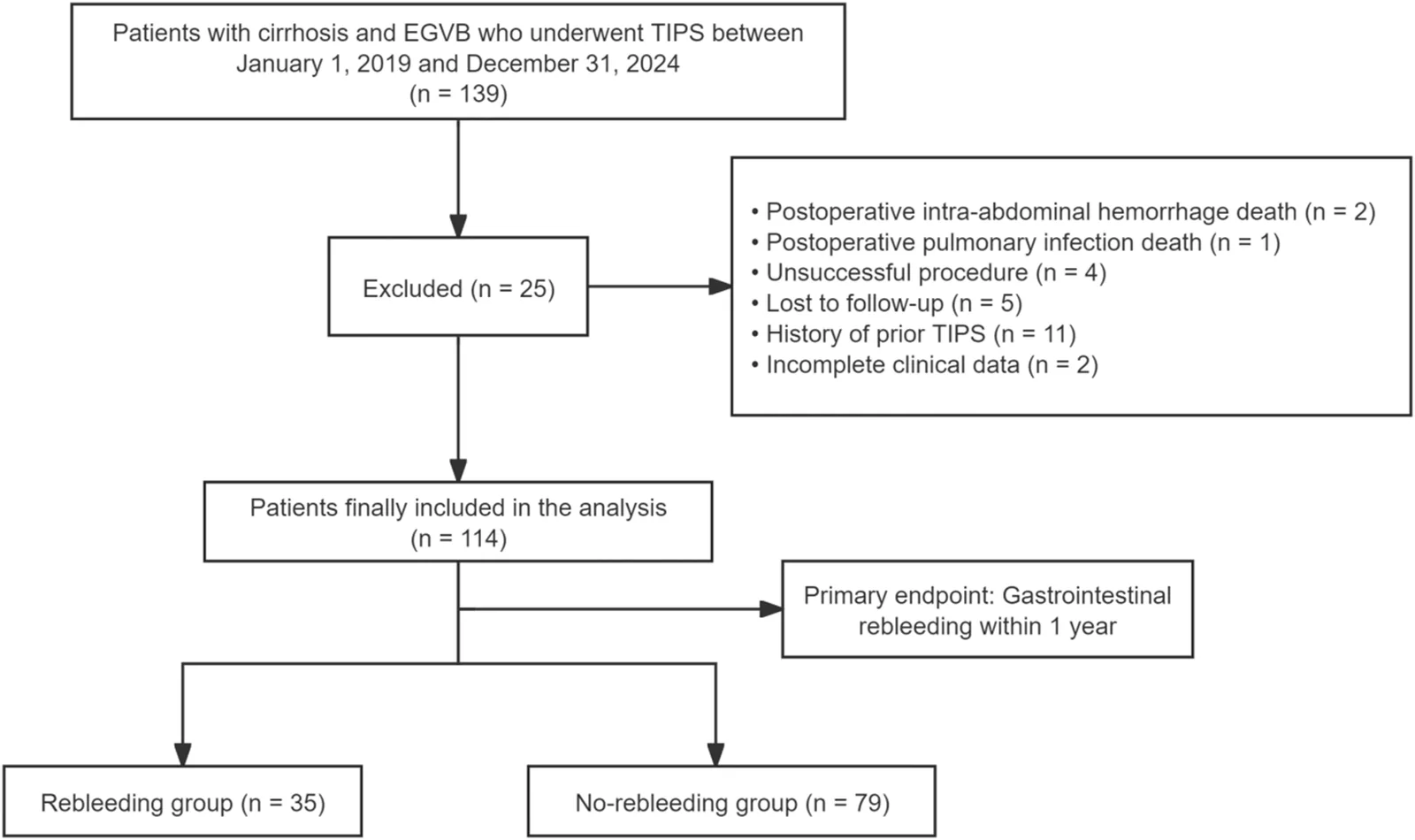
Flowchart of patient selection.

**Table 1:** Patient characteristics.

Patient characteristics	All patients (*n* = 114)	Rebleed (*n* = 35)	No-rebleed (*n* = 79)	*P*-Value	SMD
Sex, *n* (%)				0.488	0.139
Female	34 (30)	12 (34)	22 (28)		
Male	80 (70)	23 (66)	57 (72)		
Prior endoscopic treatment, *n* (%)				0.403	0.168
Yes	33 (29)	12 (34)	21 (27)		
No	81 (71)	23 (66)	58 (73)		
Age	55.8 ± 10.3	57.0 ± 11.8	55.2 ± 9.6	0.434	0.168
Etiology of Liver Cirrhosis, *n* (%)				0.224	0.351
Viral	72 (63)	18 (51)	54 (68)		
Alcohol	20 (18)	8 (23)	12 (15)		
Other	22 (19)	9 (26)	13 (17)		
Child-pugh, *n* (%)				0.751	0.208
A	24 (21)	9 (26)	15 (19)		
B	73 (64)	20 (57)	53 (67)		
C	17 (15)	6 (17)	11 (14)		
MELD	10 (9–14)	11 (9–15)	10 (9–13)	0.541	0.125
Sodium(mmol/L)	137.7 ± 4.0	136.0 ± 3.0	138.5 ± 4.1	0.001	0.705
Albumin(g/L)	31.0 ± 4.6	30.3 ± 5.0	31.2 ± 4.5	0.314	0.203
Tbil(μmol/L)	18.8 (14.8–28.9)	25.2 (16.3–33.2)	18.3 (14.5–26.2)	0.085	0.198
PT	15.0 ± 2.4	14.9 ± 2.1	15.1 ± 2.5	0.704	0.081
INR	1.33 ± 0.22	1.32 ± 0.19	1.33 ± 0.23	0.743	0.070
Creatinine(μmol/L)	67 (57.3–77.0)	67 (54.5–70.5)	69 (58.0–79.0)	0.442	0.177
ALT(U/L)	22 (16.0–36.8)	22 (16.0–41.5)	22 (16.0–35.0)	0.777	0.009
AST(U/L)	32.5 (23.0–47.0)	35 (23.5–55.0)	32 (23.0–45.0)	0.735	0.088
Blood ammonia	42.5 (27.0–71.0)	51.5 (37.3–73.8)	40.5 (26.0–65.8)	0.051	0.390
Hb(g/L)	77.7 ± 16.0	80.4 ± 15.0	76.5 ± 16.3	0.236	0.248
PLT	57 (40.3–87.8)	48 (38.0–68.0)	62 (42.0–95.0)	0.086	0.211
Diabetes, *n* (%)				0.297	0.220
Yes	23 (20)	5 (14)	18 (23)		
No	91 (80)	30 (86)	61 (77)		
Ascites, *n* (%)				0.248	0.228
Yes	92 (81)	26 (74)	66 (84)		
No	22 (19)	9 (26)	13 (16)		
Hepatic carcinoma, *n* (%)				0.395	0.179
Yes	18 (16)	4 (11)	14 (18)		
No	96 (84)	31 (89)	65 (82)		
Type of stent, *n* (%)				0.029	0.438
Covered	81 (71)	20 (57)	61 (77)		
Bare metal	33 (29)	15 (43)	18 (23)		
Stent diameter, *n* (%)				0.254	0.227
7mm	25 (22)	10 (29)	15 (19)		
8mm	89 (78)	25 (71)	64 (81)		
Combined embolization, *n* (%)				0.253	0.291
Yes	107 (94)	31 (89)	76 (96)		
No	7 (6)	4 (11)	3 (4)		
Preoperative PPG	30.1 ± 6.0	29.2 ± 5.4	30.5 ± 6.2	0.264	0.237
Postoperative PPG	15.1 ± 2.6	15.2 ± 2.2	15.1 ± 2.8	0.792	0.057
Absolute reduction in PPG	15.0 ± 4.6	14.0 ± 4.4	15.5 ± 4.7	0.106	0.338
Postoperative PPG decreased by ≥50%, *n* (%)				0.114	0.320
Yes	74 (65)	19 (54)	55 (70)		
No	40 (35)	16 (46)	24 (30)		
Shunt dysfunction, *n* (%)				<0.001	0.758
Yes	19 (17)	13 (37)	6 (8)		
No	95 (83)	22 (63)	73 (92)		
postoperative HE, *n* (%)				0.543	0.127
Yes	20 (18)	5 (14)	15 (19)		
No	94 (82)	30 (86)	64 (81)		

SMD, standardized mean difference; Tbil, total bilirubin; MELD, model for end-stage liver disease; PPG, portosystemic pressure gradient; PT, prothrombin time; INR, international normalized ratio; Hb, hemoglobin; ALT, alanine aminotransferase; AST, aspartate aminotransferase; PLT, platelets; HE, hepatic encephalopathy.

### 3.2 Risk factors for rebleeding

Univariate Cox regression analysis revealed that serum sodium (HR = 0.87, 95% CI: 0.80–0.95, *P* = 0.001), type of stent (covered vs. bare-metal, HR = 0.46, 95% CI: 0.23–0.89, *P* = 0.022), and shunt dysfunction (HR = 4.89, 95% CI: 2.45–9.78, *P* < 0.001) were significantly associated with postoperative rebleeding. Blood ammonia (HR = 1.01, 95% CI: 1.00–1.02, *P* = 0.051) showed borderline significance. Given the extreme imbalance in sample size between the combined embolization group (94%) and the non-embolization group (6%), reliable statistical inference regarding its association with rebleeding could not be made. Therefore, this variable was not included in the multivariate analysis. No significant associations were observed between rebleeding and age, prior endoscopic treatment, sex, etiology of liver cirrhosis, Child-Pugh grade, MELD score, albumin, total bilirubin, PT, INR, creatinine, ALT, AST, Hb, PLT, diabetes mellitus, ascites, hepatocellular carcinoma, stent diameter, preoperative PPG, postoperative PPG, absolute reduction in PPG, postoperative PPG reduction ≥50%, or postoperative hepatic encephalopathy (all *P* > 0.05). Detailed univariate analysis results are presented in Table 2. Variables with *P* < 0.05 in the univariate analysis (serum sodium, type of stent, and shunt dysfunction) were subsequently entered into a multivariate Cox regression model. The multivariate analysis showed that hyponatremia (HR = 0.85, 95% CI: 0.77–0.93, *P* < 0.001) and shunt dysfunction (HR = 4.90, 95% CI: 2.36–10.15, *P* < 0.001) were independent risk factors for rebleeding, whereas type of stent was no longer statistically significant in the multivariate model (HR = 1.69, 95% CI: 0.84–3.42, *P* = 0.143). The results of the multivariate analysis are presented in Table 3.

**Table 2:** Univariate Cox regression analysis for rebleeding after TIPS.

Variable	HR (95% CI)	*P*-Value
Sex(Male)	0.79 (0.39–1.59)	0.511
Prior endoscopic treatment	0.72 (0.36–1.45)	0.358
Age	1.02 (0.99–1.06)	0.208
Etiology of Liver Cirrhosis(Viral)		
Alcohol	1.48 (0.62–3.56)	0.376
Other	1.71 (0.76–3.80)	0.191
Child-pugh(A)		
B	0.66 (0.30–1.45)	0.300
C	0.85 (0.30–2.38)	0.753
MELD	1.03 (0.95–1.11)	0.499
Sodium	0.87 (0.80–0.95)	**0** **.** **001**
Albumin	0.96 (0.89–1.03)	0.269
Tbil	1.00 (0.99–1.03)	0.340
PT	0.96 (0.83–1.11)	0.608
INR	0.69 (0.14–3.35)	0.645
Creatinine	1.00 (0.99–1.01)	0.587
ALT	1.00 (0.99–1.01)	0.718
AST	1.00 (0.99–1.01)	0.970
Blood ammonia	1.01 (1.00–1.02)	0.051
Hb	1.01 (0.99–1.03)	0.276
PLT	1.00 (0.99–1.01)	0.369
Diabetes	0.70 (0.27–1.80)	0.456
Ascites	0.59 (0.28–1.25)	0.169
Hepatic carcinoma	0.75 (0.27–2.14)	0.595
Type of stent	0.46 (0.23–0.89)	**0**.**022**
Stent diameter	0.68 (0.33–1.41)	0.300
Preoperative PPG	0.97 (0.91–1.03)	0.278
Postoperative PPG	1.01 (0.90–1.14)	0.876
Absolute reduction in PPG	0.95 (0.88–1.02)	0.127
Postoperative PPG decreased by ≥50%, *n* (%)	0.62 (0.32–1.21)	0.160
Shunt dysfunction	4.89 (2.45–9.78)	**<0**.**001**
postoperative HE	0.92(0.36–2.36)	0.857

Bold *P* values indicate statistical significance (*P* < 0.05).

**Table 3:** Multivariable Cox regression for rebleeding after TIPS.

Variable	HR (95% CI)	*P*-Value
Sodium	0.85 (0.77–0.93)	**<0** **.** **001**
Type of stent	1.69 (0.84–3.42)	0.143
Shunt dysfunction	4.90 (2.36–10.15)	**<0**.**001**

Bold *P* values indicate statistical significance (*P* < 0.05).

### 3.3 Comparison of cumulative rebleeding incidence based on serum sodium level

Using a commonly used diagnostic threshold for hyponatremia in cirrhosis (21), patients were stratified into a low sodium group (<135 mmol/L) and a high sodium group (≥135 mmol/L). To validate the robustness of this cutoff, we performed a sensitivity analysis by replacing continuous serum sodium with a binary variable for hyponatremia (serum sodium < 135 mmol/L) in the multivariate Cox regression model. The results showed that hyponatremia was associated with a borderline significant increase in the risk of rebleeding (HR = 1.97, 95% CI: 1.00–3.87, *P* = 0.050). Kaplan–Meier analysis further revealed that the cumulative incidence of rebleeding within 1 year postoperatively was significantly higher in the low sodium group than in the high sodium group (log-rank test, *P* = 0.041). The divergence between the two groups became apparent at 6 months postoperatively, widened further by 9 months, and stabilized at 12 months (Figure 2).

**Figure 2:**
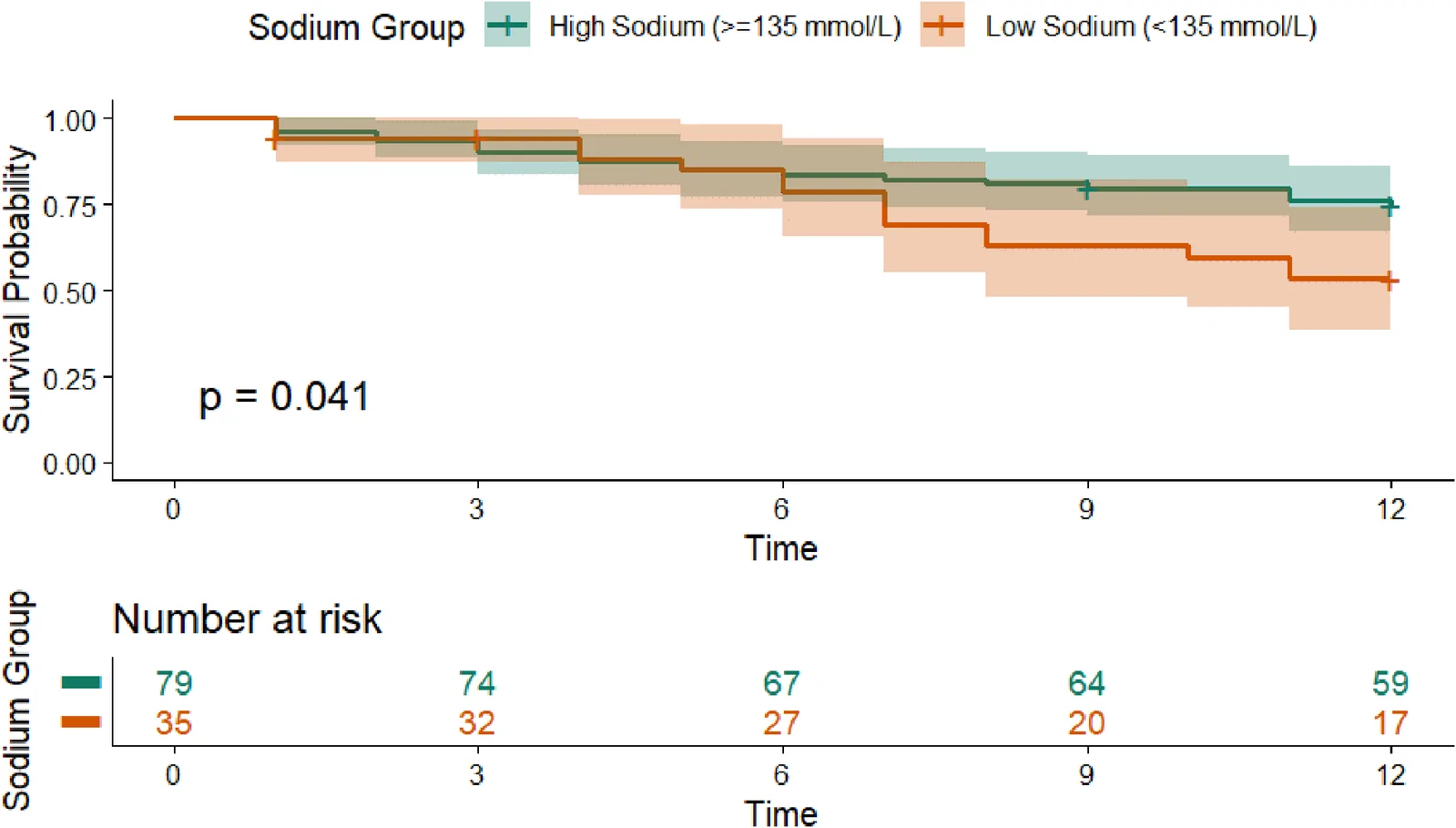
Kaplan–meier curves showing rebleeding-free survival after TIPS, stratified by serum sodium level (<135 mmol/L vs. ≥ 135 mmol/L). The low sodium group had significantly lower rebleeding-free survival than the high sodium group (log-rank *P* = 0.041). Number at risk is shown below the *x*-axis.

## 4 Discussion

Gastroesophageal variceal hemorrhage (VH) is a life-threatening complication of portal hypertension. Although transjugular intrahepatic portosystemic shunt (TIPS) achieves immediate hemostasis in 90%–100% of patients with active bleeding (8, 9), recurrent bleeding represents a persistent clinical challenge (22). Importantly, the underlying causes of recurrent hemorrhage have not been systematically elucidated in most prior reports, which limits the formulation of targeted prevention strategies. In this single-center cohort study of 114 cirrhotic patients with EGVB, we identified a 1-year rebleeding rate of 30.7%, which falls within the previously reported range of 6%–40% (13). Multivariate Cox regression analysis further revealed serum sodium and shunt dysfunction as independent risk factors, underscoring the critical roles of preoperative electrolyte optimization and vigilant postoperative shunt surveillance in risk stratification. Prior endoscopic treatment history was not significantly associated with rebleeding on univariate analysis (HR = 0.72, 95% CI: 0.36–1.45, *P* = 0.358) and was therefore not included in the multivariate model, suggesting that it does not substantially confound the observed associations.

Our study identified serum sodium as an independent risk factor for rebleeding after TIPS (HR = 0.85, 95% CI: 0.77–0.93, *P* < 0.001), consistent with previous reports. Schepke et al. (23) first established serum sodium as an independent predictor of post-TIPS survival, and Li et al. (24) subsequently developed a nomogram validating its predictive value for EGVB (*P* < 0.05). Mechanistically, hyponatremia not only serves as a serologic marker of splanchnic vasodilation, reduced effective circulating volume, and renin-angiotensin-aldosterone system activation in cirrhosis (25), but may also facilitate rebleeding by exacerbating portal pressure elevation and collateral recruitmen. Supporting evidence from composite models includes the MELD-Na score, which demonstrated good predictive performance for 6-week rebleeding after endoscopic hemostasis (AUROC 0.797) (26), and the ALTA study, which confirmed MELD-Na as an independent predictor of 1-year post-TIPS rebleeding (OR = 1.07, *P* = 0.004) (18). Given that Angeli et al. (21) reported that nearly half (49.4%) of patients with cirrhosis have serum sodium ≤135 mmol/L, and that this threshold is significantly associated with the occurrence of portal hypertensive complications, we used a cutoff value of 135 mmol/L to perform Kaplan–Meier survival analysis. The results showed that the cumulative incidence of rebleeding within 1 year postoperatively was significantly higher in the low sodium group than in the high sodium group (*P* = 0.041), confirming the value of hyponatremia in stratifying rebleeding risk. Although the binary analysis of hyponatremia yielded borderline significance (*P* = 0.050), likely attributable to the reduced statistical power from dichotomization. This does not diminish the strong predictive value of continuous serum sodium in the primary analysis (*P* < 0.001). Thus, preoperative correction of hyponatremia may be beneficial, and future studies should assess the feasibility of incorporating serum sodium into dynamic predictive models.

Shunt dysfunction emerged as the strongest independent risk factor for rebleeding (HR = 4.90, 95% CI: 2.36–10.15, *P* < 0.001). Notably, this association remained significant even when dysfunction was modeled as a time-dependent covariate using the counting process approach, a strategy that effectively eliminates immortal time bias. This finding aligns with the observations of Pitton et al. (27), who underscored its central role in long-term shunt failure and subsequent variceal rebleeding. Although stent type was significant on univariate analysis (HR = 1.69, 95% CI: 0.84–3.42, *P* = 0.143), with higher rebleeding rates in bare-metal stent recipients (43% vs. 23%, *P* = 0.029), this association lost significance after adjusting for shunt dysfunction and serum sodium (HR = 0.58, 95% CI: 0.29–1.17, *P* = 0.129). This suggests that the impact of stent type is largely mediated by shunt dysfunction, as bare-metal stents are more prone to stenosis, leading to portal pressure resurgence and rebleeding (28, 29)-a pattern also observed in the ALTA study (18). Therefore, in the contemporary era of widespread covered stent use, the direct impact of stent type on rebleeding may be diminished, warranting further validation in large-scale studies. Furthermore, neither postoperative portosystemic pressure gradient (15.2 ± 2.2 vs. 15.1 ± 2.8 mmHg, *P* = 0.792) nor a ≥ 50% reduction in PPG (54% vs. 70%, *P* = 0.114) was significantly associated with rebleeding (*P* = 0.876 and *P* = 0.160, respectively). Although a recent large-sample study (*n* = 647) identified pre-procedural PPG as an independent predictor of rebleeding in patients with patent TIPS (OR=1.182, 95%CI: 1.067-1.309), this association was not replicated in our cohort. This discrepancy may be attributable to differences in study population (our cohort included all TIPS recipients regardless of shunt patency), sample size, and the retrospective nature of both studies (30). Nonetheless, shunt dysfunction was markedly more frequent in the rebleeding group (37% vs. 8%, *P* < 0.001), indicating that even when immediate hemodynamic targets are achieved, long-term dysfunction can elevate portal pressure, refill incompletely regressed collaterals, and precipitate bleeding. Therefore, achieving intraoperative targets or using covered stents does not preclude late dysfunction,close postoperative monitoring of shunt function and timely intervention are key to reducing the risk of rebleeding.

The role of adjunctive variceal embolization remains debated. The multicenter RCT by Lv et al. (17) (*n* = 134) (*n* = 134) found no significant difference in 1-year rebleeding between TIPS with and without embolization (11.6% vs. 13.8%, *P* = 0.566), arguing against routine use. Conversely, a meta-analysis by Giri et al. (31) (11 studies, *n* = 1,044) suggested that adjunctive embolization reduced rebleeding risk (OR = 2.01, 95% CI: 1.42–2.83), albeit with moderate-quality evidence and no improvement in shunt dysfunction or mortality. In our cohort, the 94% rate of combined embolization precludes reliable inference, and therefore, our data cannot inform this controversy. Regarding blood ammonia, robust evidence linking it to post-TIPS rebleeding is scarce. Da B et al. (32) showed that an ammonia-based modified CTP score was superior to MELD and MELD-Na for predicting survival (highest C-index, *P* < 0.0001), but that study prioritized mortality and did not specifically evaluate the predictive performance of blood ammonia for rebleeding. In our study,preprocedural ammonia was numerically higher in the rebleeding group [51.5 (37.3–73.8) vs. 40.5 (26.0–65.8) μmol/L], with borderline significance on univariate analysis (*P* = 0.051), hinting at a potential association. As this variable was not entered into the multivariate model, whether blood ammonia independently predicts rebleeding warrants further investigation.

## 5 Study limitations

This study has several limitations. First, due to its single-center retrospective design and relatively small sample size (*n* = 114), the statistical power to detect associations for certain potential risk factors (e.g., blood ammonia, which showed borderline significance in univariate analysis) may have been limited, increasing the possibility of false-negative conclusions. Second, as the data were derived from a single center, and the distributions of certain variables exhibited considerable imbalance (e.g., 94% of patients underwent combined embolization and 78% received 8-mm stents), the findings warrant validation in multicenter cohorts with greater heterogeneity. Third, owing to the inherent limitations of retrospective studies, several previously established potential predictors were not included in our analysis, such as the MELD-Na score and the presence of portal hypertensive gastropathy on pre-TIPS endoscopy, which may have affected the completeness of the model. Fourth, the follow-up period was limited to 1 year postoperatively, precluding assessment of the impact of the identified risk factors on long-term rebleeding risk. Fifth, the near-universal use of combined embolization in our cohort (94%) resulted in an extreme imbalance in group sizes (94% vs. 6%). Sixth,detailed endoscopic characteristics of esophagogastric varices—including diameter, morphology, and red signs—were not uniformly documented across preoperative reports, precluding their inclusion in the regression analysis. To partially mitigate this limitation, Child-Pugh grade and portosystemic pressure gradient (PPG) were incorporated as objective surrogate markers reflecting portal hypertension severity. Nonetheless, the lack of standardized endoscopic data may have limited our ability to fully evaluate the impact of variceal severity on post-TIPS rebleeding, underscoring the need for prospective studies with systematic endoscopic reporting. Seventh,TIPS indications (emergency/salvage vs. elective secondary prophylaxis) were not included in the analysis. Although emergency TIPS is rarely performed in our center, we acknowledge that this distinction may influence rebleeding risk, and its absence represents a limitation of this retrospective study. Consequently, no reliable statistical inference could be made regarding the preventive effect of combined embolization on rebleeding. This limitation precludes any meaningful conclusion about the role of adjunctive embolization in this study. In light of these limitations, our findings require further validation through larger, multicenter, prospective studies. Future research should consider incorporating a broader range of candidate variables and extending the follow-up period to develop more robust risk prediction models.

## 6 Conclusion

Our study identified Serum sodium and shunt dysfunction as independent risk factors for recurrent gastrointestinal bleeding within 1 year after TIPS in patients with EGVB, with shunt dysfunction demonstrating the strongest association. Preoperative correction of hyponatremia and optimization of serum sodium levels, along with early detection of shunt dysfunction through regular ultrasound surveillance and timely intervention, may help reduce the risk of rebleeding and improve patient outcomes. These findings warrant further validation through large-scale, multicenter, prospective studies.

## Data Availability

The raw data supporting the conclusions of this article will be made available by the authors, without undue reservation.
